# Efficacy and safety of corticosteroids prophylaxis in cardiac surgery: A protocol for systematic review and meta-analysis

**DOI:** 10.1097/MD.0000000000023240

**Published:** 2020-12-11

**Authors:** Jian He, Yuling Zhang, Zhihuang Qiu, Tianci Chai, Guanhua Fang, Yunnan Hu, Fan Xu, Qiuyu Huang, Hui Zheng, Hao Zhou, Mengyue Tian, Liang Wan Chen

**Affiliations:** a Department of Cardiac Surgery, Fujian Medical University Union Hospital, Fuzhou; b School of Nursing, Xuzhou Medical University, Xuzhou, China.

**Keywords:** cardiac surgery, cardiopulmonary bypass, inflammation, meta-analysis, steroid

## Abstract

**Background:**

Although corticosteroid prophylaxis in adult cardiac surgery has been studied extensively for 40 years, its role remains controversial, and the optimal dose remains uncertain. The objective of this meta-analysis was to estimate the clinical benefits and risks of corticosteroid use in cardiopulmonary bypass.

**Methods:**

We will search Pubmed, Web of Science, Embase, Clinical Trials, and Cochrane Central Register of Controlled Trials for relevant clinical trials published in any language before August 1, 2020. Randomized controlled trials (RCTs) of interest which meet inclusion criteria published or unpublished will be included. We will divide the included studies into child and adult groups for analysis. If sufficient data are available, the included trials will be divided into 4 subgroups: ≤20 mg/kg (low dose), 20–40 mg/kg (slightly high dose), 40–100 mg/kg (high dose), and >100 mg/kg (ultra high dose) based on the equivalent hydrocortisone dose. INPLASY registration number: INPLASY2020100044.

**Results:**

The results of this study will be published in a peer-reviewed journal.

**Conclusion:**

This study will compare the efficacy of tprophylactic corticosteroids for adults and children undergoing cardiac surgery with CPB. Due to the nature of the disease and intervention methods, randomized controlled trials may be inadequate, and we will carefully consider inclusion in high-quality, non-randomized controlled trials, but this may result in high heterogeneity and affect the reliability of the results.

## 1 Introduction

Most cardiac operations are performed under cardiopulmonary bypass, however, it is well known that cardiopulmonary bypass often causes systemic inflammatory response syndrome (SIRS).^[1]^ SIRS related to complement, platelets, neutrophils, monocytes, macrophages activation and cascade (coagulation, fibrinolytic, stimulating peptide enzyme), leading to endothelial permeability increase, blood vessels and organ parenchyma cell injury, and liver, kidney, nervous system dysfunction, myocardium injury and infarction, respiratory failure, multiple organ dysfunction, and death are closely related.^[2–7]^

Corticosteroids is a low-cost drug that can effectively inhibit inflammation, limit systemic capillary leakage syndrome and reduce organ damage, thus providing a theoretical basis for its clinical application.^[8–10]^ However, corticosteroids may have their own side effects, causing hyperglycemia, which is associated with immunosuppression and poor wound healing. In addition, high doses of corticosteroids were associated with an increased risk of gastrointestinal bleeding and myocardial infarction.^[11–12]^ The beneficial effects of glucocorticoids on adults and children undergoing heart surgery remain controversial.^[13–15]^

Three meta-analysis of small RCTs showed that prophylactic corticosteroids can reduce the risk of atrial fibrillation after cardiac surgery in adults, reduce the duration of mechanical ventilation and hospital stay, but can cause some potential side effects.^[5–7]^ None of the 3 studies analyzed pediatric studies, and there was a lack of high-quality, large-sample randomized controlled trials. The clinical results of the analysis were not comprehensive and the evidence obtained was not robust. Subsequently, 2 large multicentre randomized controlled trials showed that corticosteroid treatment had no benefit in adult patients undergoing heart surgery and increased the risk of myocardial infarction.^[13–14]^ However, guidelines for adult cardiac surgery do not recommend routine prophylactic use of corticosteroids to reduce complications. Therefore, the purpose of this study was to systematically review and meta-analyze the dose-dependent benefits and risks of prophylactic glucocorticoids in adults and children undergoing cardiopulmonary bypass.

## 2 Objective

We will conduct a systematic review and meta-analysis to estimate the clinical benefits and risks of corticosteroid use in cardiopulmonary bypass.

## 3 Methods

This protocol is performed adhere to the Preferred Reporting Items for Systematic Review and Meta-Analysis Protocols (PRISMA-P) statement. The results of this systematic review and meta-analysis will be published with reference to the Preferred Reporting Items for Systematic Reviews and Meta-Analyze (PRISMA) guidelines.

### 3.1 Patient and public involvement

This study will be based on published or unpublished studies and records and will not involve patients or the public directly.

### 3.2 Eligibility criteria

#### 3.2.1 Types of studies

Only randomized controlled clinical trials comparing corticosteroid with placebo or equal volume of normal saline, initiated either before or at the time of cardiopulmonary bypass were included. Studies that used unequal concurrent medical therapies or studies that evaluated corticosteroid in off-pump cardiac surgery were excluded.

#### 3.2.2 Types of participants

Patients with heart, valve, or aortic disease are treated surgically under extracorporeal circulation and there will be no restrictions on sex, ethnicity, economic status, and education.

#### 3.2.3 Types of interventions

Cardiac surgery with cardiopulmonary bypass with or without prophylactic corticosteroid administration. For comparator study arms, trials with concomitant study arms on other interventions were not excluded, as long as patients in the comparator arm received the same treatment as the corticosteroid arm except for corticosteroid administration.

#### 3.2.4 Types of outcome measures

##### 3.2.4.1 Primary outcomes

Composite end-point, consisting of the following:

- all-cause mortality (in-hospital);
- occurrence of atrial fibrillation (in the postoperative period);
- fatal and non-fatal myocardial infarction (defined as: ECG changes, echocardiological changes, disproportionate elevation of troponines, specific biological marker);
- pulmonary complications (including pulmonary edema and/or infection);
- kidney injury (renal failure, acute renal failure, acute kidney disease, renal complications).

##### 3.2.4.2 Secondary outcomes

- postoperative infection
- neurological complications
- gastro-intestinal bleeding
- postoperative insulin use
- mechanical ventilation time
- Delirium
- length of ICU stay
- LOS hospital
- Duration of CPB
- vaso-active medication
- re-thoracotomy
- inotropic score
- blood transfusion
- re-intubation
- CRP/IL-6/IL-8 concentrations at 24 hours after cardiopulmonary bypass

### 3.3 Information sources

Two reviewers (CTC, QZH) will search Pubmed, Web of Science, Embase, and Clinical Trials, and Cochrane Central Register of Controlled

Trials for relevant clinical trials published before August 1, 2020 without any language restrictions.

### 3.4 Search strategy

The subject terms and keywords corresponding to Medical Subject Heading (MeSH) terms will be used to search for eligible trials in the databases as mentioned above with no language restrictions. Search strategies in PubMed are shown in Table 1.

**Table 1:** PubMed search strategies.

Query	Search term
#1	(((Corticosteroids [MeSH Terms]) OR ((Hormones, Adrenal Cortex [Title/Abstract]) OR (Corticosteroids [Title/Abstract])) OR (Corticoid[Title/Abstract])) OR (Corticoids [Title/Abstract])) OR (Metacortandracin [Title/Abstract])) OR (Glucocorticoids [Title/Abstract])) OR (17-Ketosteroids [Title/Abstract])) OR (Catatoxic Steroids [Title/Abstract])) OR (Steroids, Catatoxic [Title/Abstract])) OR (Steroids[Title/Abstract])) OR (Dehydrocortisone [Title/Abstract])) OR (Delta-Cortisone [Title/Abstract])) OR (Metacortandracin [Title/Abstract])) OR (Prednison [Title/Abstract])) OR (Meprednisone [Title/Abstract])) OR (Alpha-methylprednisolone [Title/Abstract])) OR (Methylprednisolone [Title/Abstract])) OR (Metipred [Title/Abstract])) OR (6-Methylprednisolone [Title/Abstract])) OR (6 Methylprednisolone [Title/Abstract])) OR (Urbason [Title/Abstract])) OR (Medrol[Title/Abstract])) OR (Cortisol[Title/Abstract])) OR (Hydrocortisone[Title/Abstract])) OR (Hydrocortiso [Title/Abstract])) OR (Epicortisol [Title/Abstract])) OR (Cortifair [Title/Abstract])) OR (Cortril [Title/Abstract])) OR (Methylfluorprednisolone [Title/Abstract])) OR (Hexadecadrol [Title/Abstract])) OR (Decameth [Title/Abstract])) OR (Decaspray [Title/Abstract])) OR (Dexasone [Title/Abstract])) OR (Dexpak [Title/Abstract])) OR (Maxidex [Title/Abstract])) OR (Millicorten[Title/Abstract])) OR (Oradexon [Title/Abstract])) OR (Hexadrol [Title/Abstract])) OR (Anti-inflammator [Title/Abstract])) OR (Anti next inflammatory [Title/Abstract])) OR (Antiinflammator [Title/Abstract])) OR (Antiflogistic [Title/Abstract])))
#2	((Extracorporeal Circulations [MeSH Terms]) OR ((((((((((((((((((((((((Circulation, Extracorporeal [Title/Abstract]) OR (Circulations, Extracorporeal [Title/Abstract])) OR (Extracorporeal Circulations [Title/Abstract])) OR (Heart-Lung Bypass [Title/Abstract])) OR (Bypass, Heart-Lung [Title/Abstract])) OR (Bypasses, Heart-Lung [Title/Abstract])) OR (Heart Lung Bypass [Title/Abstract])) OR (Heart-Lung Bypasses [Title/Abstract])) OR (Bypass, Cardiopulmonary [Title/Abstract])) OR (Bypasses, Cardiopulmonary [Title/Abstract])) OR (Cardiopulmonary Bypasses [Title/Abstract])) OR (Oxygenation, Extracorporeal Membrane [Title/Abstract])) OR (Extracorporeal Membrane Oxygenations [Title/Abstract])) OR (Membrane Oxygenation, Extracorporeal [Title/Abstract])) OR (Membrane Oxygenations, Extracorporeal [Title/Abstract])) OR (Oxygenations, Extracorporeal Membrane [Title/Abstract])) OR (Bypass, Left Heart [Title/Abstract])) OR (Bypasses, Left Heart [Title/Abstract])) OR (Heart Bypasses, Left [Title/Abstract])) OR (Left Heart Bypasses [Title/Abstract])) OR (Heart Bypasses [Title/Abstract])) OR (Left Heart Bypass [Title/Abstract])) OR (CPB [Title/Abstract])) OR (Coronary Artery Bypass [Title/Abstract]))))
#3	(randomized controlled trial [Publication Type] OR randomized [Title/Abstract] OR placebo [Title/Abstract])
#4	#1 AND #2 AND #3

### 3.5 Data collection and analysis

We will adopt the methods described in the Cochrane Handbook for Systematic Reviews of Interventions to pool the evidence.

#### 3.5.1 Study selection

Two authors (CTC, QZH) will screen independently each title and abstract of all the papers searched and the trials do not meet the inclusion criteria described in this protocol will be excluded. Full text of all the possible eligible trials will be screened independently and in duplicate by the 2 authors. Trials which are irrelevant or do not meet the inclusion criteria will be excluded. Trials that meet the inclusion criteria and excluded studies with the reasons for their exclusion will be documented by 2 authors (CTC, QZH). If there is a disagreement between the 2 authors, we will resolve the disagreement by discussing with the third author (LYM). If necessary, we will consult the fourth author (CLW) to resolve the disagreement. Selection process will be shown in PRISMA flow chart in details.

#### 3.5.2 Data extraction and management

We will extract the following data from the trials included.

- Study characteristics: author, publication date, country, study design, randomization, periods of data collection, follow-up duration, withdrawals, and overall duration of study.
- Population characteristics: age, sex, BMI, operation, blood pressure, history of diabetes, performance status, ethnicity, history of smoking, and inclusion criteria.
- Interventions: The types, doses, time and routes of corticosteroids used in extracorporeal circulation.
- Outcomes: mortality; occurrence of atrial fibrillation; myocardial infarction; pulmonary complications; acute kidney injury; postoperative infection; postoperative insulin use; gastro-intestinal bleeding; re-thoracotomy; neurological complications; inotropic use; blood transfusion; mechanical ventilation time; re-intubation; length of ICU stay; CRP/IL-6/IL-8 concentrations at 24 hours after cardiopulmonary bypass; vaso-active medication.

We will use the pre-designed table to record the data extracted from the included trials. If relevant data of the trials is lost or unclear, we will consult the author via email before determining whether the study is included.

### 3.6 Assessment of risk of bias

The Cochrane Handbook for Systematic Reviews of Interventions will be used to assess the risk of bias of each trial included. The 2 authors (CTC, QZH) will evaluate the risk of bias based on the following domains: random sequence generation (selection bias), allocation concealment (selection bias), blinding of participants and personnel (performance bias), blinding of outcome assessment (detection bias), incomplete outcome data (attrition bias), selective outcome reporting (reporting bias), and other bias. The risk of bias in each domain will be assessed as high, low, or uncertain and the results of the evaluation will be shown on the risk of bias graph.

### 3.7 Data analysis

We will use Review Manager and Stata software to synthesise the data extracted. If the data extracted from the included studies are evaluated as highly homogeneous, we will conduct meta-analysis on them for the purpose of obtaining a clinically meaningful result. In order to carry out a standard meta-analysis, we will use the Chi^2^ and *I*^2^ statistic test to evaluate statistical heterogeneity among the studies. If there is high heterogeneity (*P* < .1 or *I*^2^ statistic >50%), we will use the DerSimonian and Laird random effect model to analyze the extracted data. Otherwise, we will adopt fixed-effect model to analyze the data. We will adopt the Mantel-Haenszel method to pool the binary data and the results will be reported in the form of relative risk (RR) with the 95% confidence interval (CI). Inverse variance analysis method will be used to pool the continuous data and the results will be reported in the form of standardized mean difference (SMD) with 95% confidence interval (CI).

#### 3.7.1 Subgroup analysis

If there is substantial heterogeneity and the available data are sufficient, we will perform subgroup analysis for searching potential origins of heterogeneity. If sufficient data are available, the included trials will be divided into 4 subgroups: ≤20 mg/kg (low dose), 20–40 mg/kg (slightly high dose), 40–100 mg/kg (high dose), and >100 mg/kg (ultra high dose) based on the equivalent hydrocortisone dose.

#### 3.7.2 Sensitivity analysis

We will conduct sensitivity analysis to evaluate the robustness and the reliability of aggregation results by eliminating trials with high bias risk.

If reporting bias exists, we will use the methods of fill and trim to analyze publication bias.

### 3.8 Publication bias

Funnel charts and Eggers test will be adopted to assess publication bias if there are no less than 10 eligible trials. If reporting bias is suspected in a trial, we will contact the corresponding author via email to find out whether there are additional outcome data which were not reported.

### 3.9 Evidence evaluation

We will classify the quality of all evidence into 4 levels (high, medium, low, and very low) in accordance with the criteria of GRADE (study limitations, imprecision, publication bias, indirectness bias, and effect consistency).^[17]^

## 4 Discussion

Systemic inflammatory response in the incidence of complications after heart surgery plays a crucial role in elevated levels of proinflammatory cytokines and complement activation leads to the occurrence of capillary leak syndrome,^[1]^ thereby worsening organ function.^[2–7]^ Corticosteroids can effectively inhibit systemic inflammation and reduce the level of inflammatory factors, providing a theoretical basis for prophylactic administration in cardiac surgery under CPB.^[8–10]^

A large number of randomized controlled trials showed that the prophylactic use of corticosteroids in patients with heart operation under CPB did not bring any benefits, but increased the risk of myocardial infarction, prolonged the time of mechanical ventilation.^[10,13,14,18]^ Adult cardiac surgery guidelines and clinical routine cardiac surgery in adults and children are not used in preventive corticosteroids. However, the clinical effects of corticosteroids are dose-dependent. The strength of its anti-inflammatory effects and clinical side effects are closely related to corticosteroids dosage.^[3,4,7]^ We assumed that there is an appropriate dosage range that can effectively inhibit systemic inflammation and trigger protective function of corticosteroids. Corticosteroids have the least side effect, protecting rather than destroying cardiomyocytes. Therefore, we are going to conduct a systematic review and meta-analysis of dose-dependent benefits and risks of corticosteroid prevention in cardiopulmonary surgery.

## Author contributions

Liang Wan Chen and Zhihuang Qiu are the guarantor of the article. Jian He and Yuling Zhang conceived and designed the study. Zhihuang Qiu, Tianci Chai, Guanhua Fang, Yunnan Hu, Fan Xu, Qiuyu Huang, and Fei Luo drafted this protocol. Peipei Zhang, Tianci Chai, Hui Zheng, Hui Zheng, Hao Zhou, and Mengyue Tian, will perform the search, screening and extraction. Liang Wan Chen and Zhihuang Qiu have strictly reviewed this protocol and approved of publication.

**Conceptualization:** Jian He, Yuling Zhang, Guanhua Fang.

**Data curation:** Jian He, Yuling Zhang, Guanhua Fang, Yunnan Hu.

**Formal analysis:** Jian He, Yuling Zhang, Hao Zhou.

**Funding acquisition:** Mengyue Tian, Liangwan Chen.

**Investigation:** Jian He, Fan Xu.

**Methodology:** Jian He.

**Project administration:** Tianci Chai, Qiuyu Huang.

**Resources:** Jian He, Tianci Chai, Hui Zheng.

**Software:** Jian He.

**Supervision:** Zhihuang Qiu.

**Validation:** Jian He, Zhihuang Qiu.

**Visualization:** Jian He, Zhihuang Qiu.

**Writing – original draft:** Jian He, Zhihuang Qiu.

**Writing – review & editing:** Jian He.
